# Biological effects 26 years after simulated deep-sea mining

**DOI:** 10.1038/s41598-019-44492-w

**Published:** 2019-05-29

**Authors:** Erik Simon-Lledó, Brian J. Bett, Veerle A. I. Huvenne, Kevin Köser, Timm Schoening, Jens Greinert, Daniel O. B. Jones

**Affiliations:** 1 National Oceanography Centre, Empress Dock, SO14 3ZH Southampton, UK; 2 Ocean and Earth Science, University of Southampton, SO14 3ZH Southampton, UK; 3 GEOMAR Helmholtz Centre for Ocean Research Kiel, D-24148 Kiel, Germany; 4 Christian-Albrechts University Kiel, Institute of Geosciences, D-24098 Kiel, Germany

**Keywords:** Marine biology, Community ecology, Ecosystem ecology, Conservation biology, Biodiversity

## Abstract

The potential for imminent abyssal polymetallic nodule exploitation has raised considerable scientific attention. The interface between the targeted nodule resource and sediment in this unusual mosaic habitat promotes the development of some of the most biologically diverse communities in the abyss. However, the ecology of these remote ecosystems is still poorly understood, so it is unclear to what extent and timescale these ecosystems will be affected by, and could recover from, mining disturbance. Using data inferred from seafloor photo-mosaics, we show that the effects of simulated mining impacts, induced during the “DISturbance and reCOLonization experiment” (DISCOL) conducted in 1989, were still evident in the megabenthos of the Peru Basin after 26 years. Suspension-feeder presence remained significantly reduced in disturbed areas, while deposit-feeders showed no diminished presence in disturbed areas, for the first time since the experiment began. Nevertheless, we found significantly lower heterogeneity diversity in disturbed areas and markedly distinct faunal compositions along different disturbance levels. If the results of this experiment at DISCOL can be extrapolated to the Clarion-Clipperton Zone, the impacts of polymetallic nodule mining there may be greater than expected, and could potentially lead to an irreversible loss of some ecosystem functions, especially in directly disturbed areas.

## Introduction

Abyssal polymetallic nodule mining has attracted considerable scientific and public attention^1–3^. The impacts of mining are likely to extend over extremely large areas^4^ and lead to major changes in the benthic fauna^5^, some of which may be long-lasting^6^. Polymetallic nodule fields are an unusual mosaic habitat where the hard substratum provided by nodules combined with the background sediment increases habitat complexity^7,8^ and promotes the development of diverse benthic communities^9–11^. Commercially viable areal densities of nodules are reported to occur in the Mid-Indian Ocean basin, around the Cook Islands (equatorial Pacific), in the Clarion-Clipperton Zone (CCZ; NE Pacific), and in the Peru Basin (SE Pacific)^12^. Although the potential effects of mining have been investigated for decades^13,14^, the ecology of these remote areas is still poorly understood. To what extent and timescale these ecosystems would be affected by, and could recover from, mining disturbance remains unclear.

In addition to the likely direct mortality of benthic fauna along mining machine tracks, nodule removal will also alter the character of the seafloor habitat for the very long-term (i.e. thousands of years)^15^. The hard substratum provided by nodules is a basic requirement for many attached sessile organisms, and for smaller motile fauna that inhabit nodule crevices^16–18^. Nodule-attached taxa can represent 60–70% of the total numerical abundance of fauna present in polymetallic nodule fields^7,11^. Consequently, mass removal of nodules is likely to have a substantial impact on local and regional biodiversity metrics^8,19^. The mining process is also expected to disturb (completely remove) and re-suspend the upper 10–15 cm of the sediment column^4^. The resultant re-suspended sediment plumes and their subsequent resedimentation will likely affect the feeding of suspension feeders, and potentially limit the recolonization of disturbed areas by affecting larval dispersal, mortality and settlement success^6^. The physical and chemical alteration of the surface sediment environment is likely to be long-lasting (>20 years), even in areas affected by resedimentation alone^20^.

**Figure 1:**
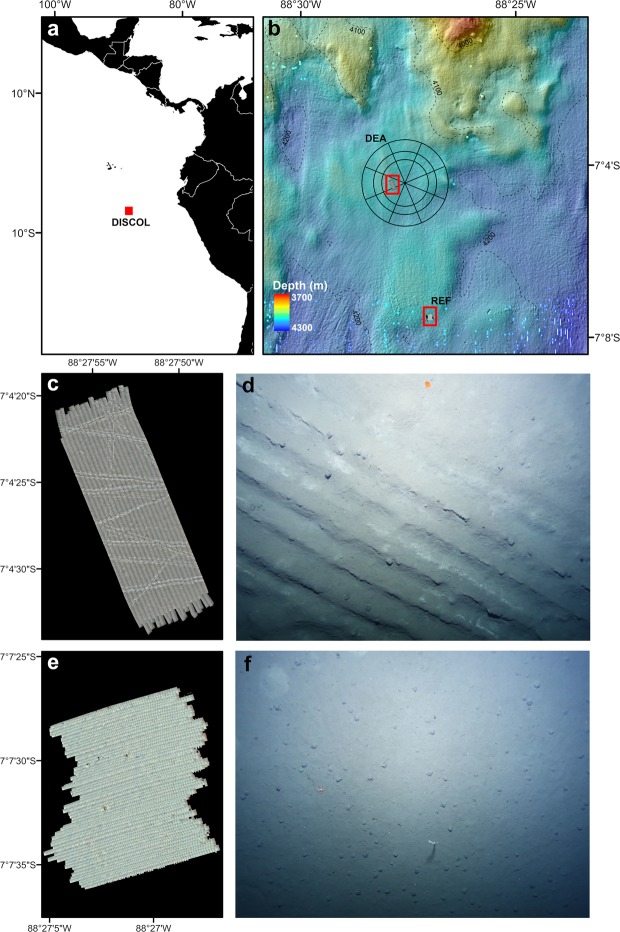
Study locations and seafloor imagery collected at the Peru Basin. (**a**) General location of DISCOL experimental area in the Peru Basin (red square). (**b**) Locations of the two investigated areas, one within the DEA and one 3.5 km south (open red squares). Full mosaics produced for the DISCOL Experimental Area site (**c**) and the REF site (**e**) study areas. (**d**) Example image of plough-disturbed seafloor from the DEA (area: 62.4 m^2^). (**f**) Example seafloor image from the, undisturbed, REF site (area: 71.3 m^2^). Maps were generated using ArcMap software v10.2^52^.

Several deep-sea mining simulation studies have been carried out to investigate biological effects^5^. The “DISturbance and reCOLonization experiment” (DISCOL), conducted in the Peru Basin in 1989 (Fig. 1), is the largest disturbance experiment carried out to date^14^. An 8 m-wide plough-harrow was towed 78 times through the centre of a study area (~1100 ha), the DISCOL Experimental Area (DEA), to simulate some of the impacts expected from the use of a nodule collector vehicle^14,21^. Physical disturbance of two forms resulted: (i) within the plough tracks (PTs), most polymetallic nodules were buried and the surface sediment structure became a mosaic of clasts of previously buried consolidated ‘clay’ and flocculent, redeposited material with a high water content^14^, and (ii) unploughed areas, which were subject to sediment redeposition. About 20% of the DEA was directly ploughed, the remainder was blanketed in a redeposited sediment layer up to 30 mm-thick^22^. Four areas located 3–4 km from the DEA, and presumed beyond the influence of the redeposition, served as reference sites during the investigations that monitored the recolonization patterns of the DISCOL area, 0, 0.5, 3, and 7 years after the disturbance^23–26^.

**Figure 2:**
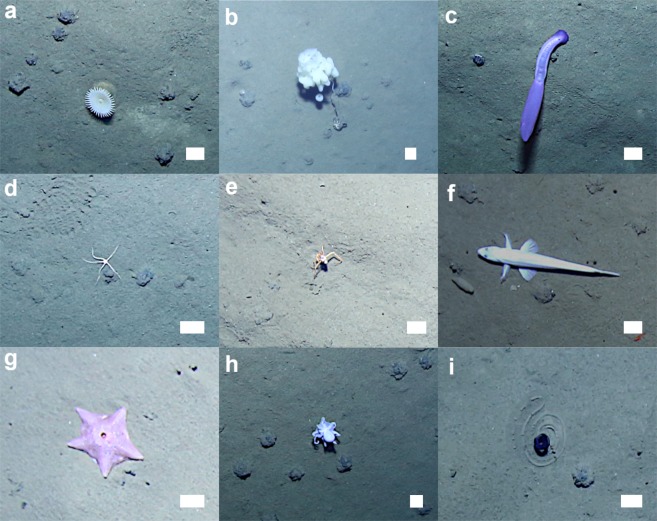
Examples of megafauna photographed at the Peru Basin seafloor during AUV survey. (**a**) Anthozoa, Actiniaria msp-2. (**b**) Porifera, Aphrocallistidae msp-1. (**c**) Holothuroidea, *Psychropotes longicauda* -violet morphotype-. (**d**) Ophiuroidea, Ophiuroid msp-1. (**e**) Crustacea, *Probeebei mirabilis*. (**f**) Actinopterygii, *Bathysaurus mollis*. (**g**) Asteroidea, Hymenaster msp-3. (**h**) Cephalopoda, ‘Casper’ msp. (**i**) Enteropneusta, *Tergivelum* sp. inc. Scale bars represent 5 cm. See Table S1 for further taxonomical detail.

Simulated mining in the DEA appeared to produce changes in the benthic community structure, with varying effects among the size classes and functional groups of the fauna (e.g.)^23–25^. Megafauna are the largest animals (>1 cm length; e.g. Fig. 2) inhabiting deep-sea benthic ecosystems and have hence previously been investigated using image surveys in the DISCOL site^24^. Metazoan megafauna numerical density and total taxon richness were dramatically reduced in the ploughed areas of the DEA immediately after disturbance and remained substantially reduced 7-years later; the effects being most marked in the case of nodule-attached fauna^24^. However, previous studies of impact at DISCOL have focussed on temporal change – and in part by reference to undisturbed ‘controls’ – these have, to some extent been limited by errors, variable methodology and unrelated natural variation in time^24,27^. Here we focus on a detailed spatial study implemented with a consistent methodology that enables the determination of both physical seabed disturbance level and ecological condition at metre-scale over substantial spatial extents (multi-hectare).

We revisited the DISCOL site 26 years after the mining simulation experiment to investigate whether past disturbance still influences the distribution of megabenthic faunal assemblages within this area. Complete-coverage seafloor photo-mosaics (encompassing an area of 11 ha) were generated from autonomous vehicle (AUV) imagery to simultaneously determine the present level of seafloor disturbance and link this to the spatial distribution of megafauna. We assessed the temporal and spatial responses to disturbance of dominant megafauna groups, as well as community diversity. These results are used to understand better the context and scale of potential commercial mining disturbance.

## Results

### Standing stocks

The total numerical density of megafauna exhibited statistically significant variation between the four disturbance levels considered (F_[3,21]_ = 23.5, p < 0.0001), being broadly comparable in plough tracks (PTs, level-A) and the in Southern reference area (REF, level-D) at c. 600 ind ha^−1^. Metazoan density was significantly higher (pairwise comparisons, p < 0.05) in level-B and level-C (1–10 m, and 10–50 m from PTs, respectively) than in both level-A and level-D, at c. 800 ind ha^−1^ (Table 1). Among the six dominant taxonomic groups selected for individual assessment (Fig. 2a–f), only the Holothuroidea exhibited no significant variation in density between disturbance levels (F_[3,21]_ = 1.81, p = 0.1771); the other five taxa groups assessed (i.e. Anthozoa, Porifera, Crustacea, Ophiuroidea, Fish) exhibited statistically significant density variations (F_[3, 21]_ ≥ 9.93, p < 0.0005) of substantial magnitude (η^2^ ≥ 0.57; Table 1) across the different seabed disturbance levels surveyed.

**Table 1:** Variations in megafauna standing stock and diversity by disturbance level.

	Distance from plough tacks								F_[3,21]_	p	η^2^
	0–1 m		1–10 m		10–50 m		>3500 m				
**Standing stock (ind ha** ^**−1**^ **)** ^**a**^											
Total megafauna	649	(603, 696)	**790**	(740, 842)	**798**	(747, 850)	592	(561, 624)	23.5	<0.0001	0.770
Deposit feeder	**443**	(409, 480)	**471**	(435, 509)	**470**	(435, 508)	316	(295, 337)	29.6	<0.0001	0.809
Predator/scavenger	**141**	(128, 155)	**161**	(146, 176)	**159**	(145, 174)	115	(106, 124)	14.7	<0.0001	0.677
Suspension feeder	**63.4**	(50.7, 78.2)	158.3	(137.5, 181.)	168.0	(146.6, 191.4)	161.7	(146.7, 177.7)	28.5	<0.0001	0.803
Anthozoa	**18.9**	(13.1, 26.1)	**32.6**	(24.8, 41.8)	**42.3**	(33.3, 52.7)	63.4	(55.4, 72.2)	21.1	<0.0001	0.751
Porifera	**12.6**	(7.8, 19.0)	69.1	(56.8, 83.1)	62.3	(50.6, 75.6)	58.0	(49.9, 66.9)	26.4	<0.0001	0.790
Holothuroidea	185	(159, 214)	165	(141, 192)	178	(152, 206)	151	(134, 169)	1.81	0.1771	0.205
Ophiuroidea	**227**	(209, 247)	**273**	(253, 295)	**258**	(238, 279)	136	(126, 147)	69.0	<0.0001	0.908
Crustacea	**92.6**	(80.1, 106.3)	71.4	(60.5, 83.6)	55.4	(45.9, 66.2)	56.9	(49.9, 64.4)	9.93	0.0003	0.586
Actinopterygii	**17.7**	(12.8, 23.7)	**45.7**	(37.5, 55.0)	**63.4**	(53.7, 74.3)	29.1	(24.5, 34.3)	25.8	<0.0001	0.786
**Diversity (number of taxa in c. 3500 m** ^**−2**^ **)** ^**b**^											
Richness	43.4	(39.8, 47.0)	51.0	(47.4, 54.6)	51.8	(48.2, 55.4)	47.7	(45.2, 50.2)	4.38	0.0153	0.385
Exp[H’]	**18.4**	(16.3, 20.5)	**21.6**	(19.4, 23.7)	**22.3**	(20.2, 24.4)	27.5	(26.0, 29.0)	17.8	<0.0001	0.718
1/D	**7.11**	(5.91, 8.31)	**7.55**	(6.35, 8.75)	**8.41**	(7.21, 9.61)	13.37	(12.53, 14.22)	35.3	<0.0001	0.834

**Figure 3:**
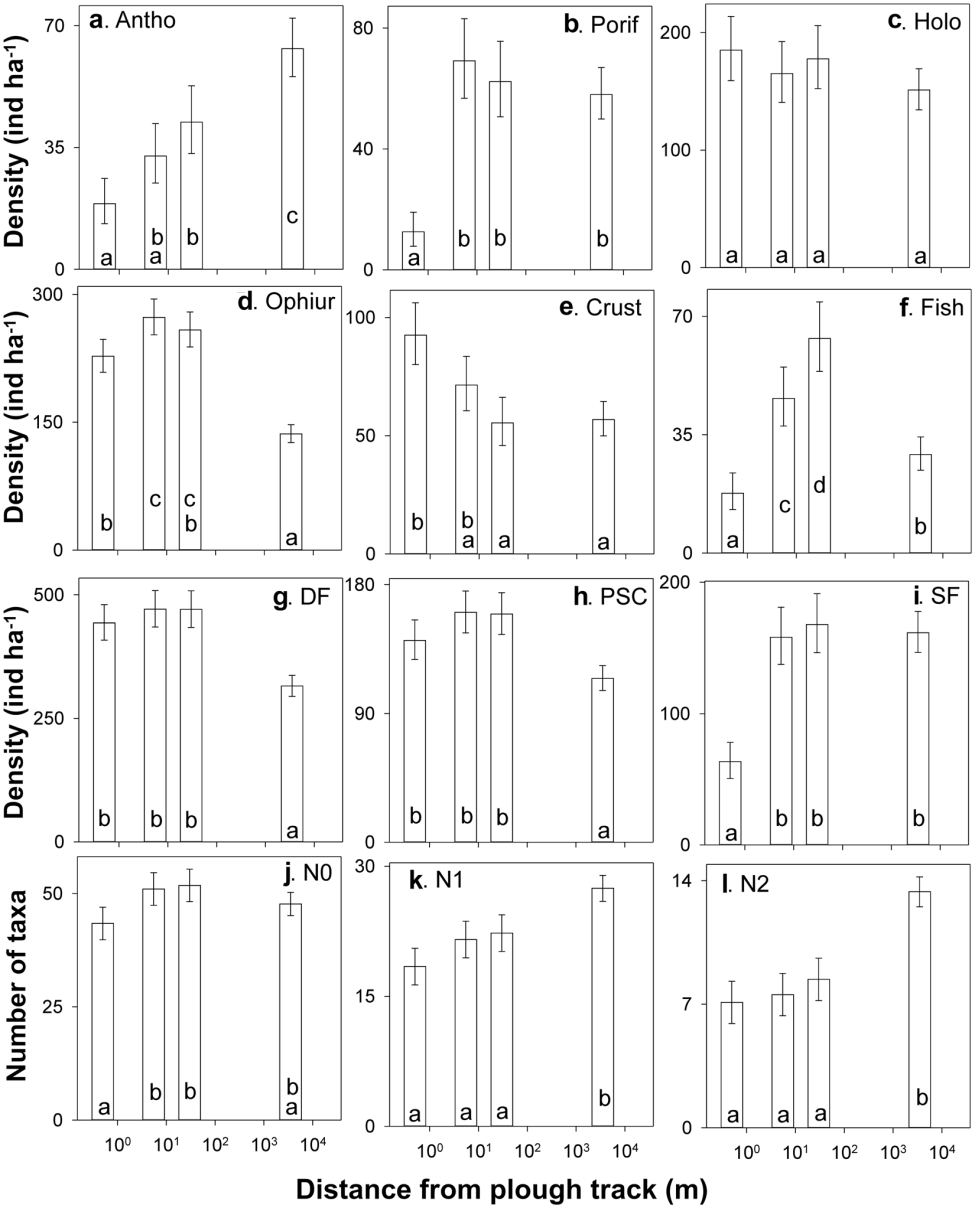
Variations in megafauna standing stock and diversity by disturbance level. (**a**–**f**) Density of selected taxa. (**g**–**i**) Density of different feeding groups. (**j**–**l**) Total megafauna diversity. Mean values across the replicate sample sets surveyed in each disturbance level are shown along with 95% confidence intervals (error bars). (**a**) Anthozoa. (**b**) Porifera. (**c**) Holothuroidea. (**d**) Ophiuroidea. (**e**) Crustacea. (**f** ) Actinopterygii. (**g**) Deposit feeders. (**h**) Predators & scavengers. (**i**) Suspension feeders. (**j**) Taxon richness. (**k**) Diversity as exp[H′]. (**l**) Diversity as 1/D. Disturbance levels as distance from plough tracks: level-A, 0–1 m; level-B, 1–10 m; level-C, 10–50 m; level-D, >3500 m. Results of pairwise comparisons are summarised as letter codes on each bar, means that do not share a common letter are statistically significantly different (p < 0.05).

Tabulated as mean (and 95% confidence interval) for disturbance levels represented by distance from plough tracks: level-A, 0–1 m; level-B, 1–10 m; level-C, 10–50 m; level-D, >3500 m. Statistical significance of variation by disturbance level is indicated by F-value and associated p-value of corresponding generalised linear model. The effect size measure η^2^ is similarly tabulated. Mean values shown in bold are those that exhibit statistically significant differences (p < 0.05) from the Southern reference area (>3500 m) in pairwise comparisons (see Fig. 3 for further details).

^a^Quasi-Poisson error; ^b^Gaussian error.

Patterns of variation with disturbance level in those five taxa showed a range of responses. Anthozoa showed a declining density with presumed intensity of disturbance (i.e. from level-D to level-A; Fig. 3a). Crustacea showed the opposed pattern; an increasing density with disturbance (Fig. 3e). Porifera density showed a dramatic step-down change in plough tracks (i.e. in level-A; Fig. 3b). Fish density was enhanced at intermediate disturbance levels (i.e. in levels-C-B; Fig. 3f), while Ophiuroidea density described a step-up change from REF (level-D) to DEA (levels-A-C) (Fig. 3d). Note that the statistical significance (p < 0.05) of individual pairwise comparisons between disturbance levels is indicated by letter code on each of the Fig. 3 plots. When assessed for the total assemblage, statistically significant variations between disturbance levels were detected for all three feeding groups (F_[3,21]_ ≥ 14.7, p < 0.0001; Table 1). Deposit feeders and predators & scavengers were uniformly enhanced in DEA relative to REF (Fig. 3g,h). In contrast, suspension feeders were substantially reduced in plough tracks relative to their essentially uniform density at lower disturbance intensities (levels-B-D; Fig. 3i).

### Assemblage diversity and composition

All of the metrics of megafauna diversity calculated exhibited statistically significant variation between disturbance levels (Table 1), however, the effect was modest in the case of taxon richness (F_[3,21]_ = 4.38, p = 0.0153, η^2^ = 0.385), and rather more substantial in the case of exponential Shannon and inverse Simpson’s indices (F_[3,21]_ ≥ 17.8, p < 0.0001, η^2^ ≥ 0.718). Taxon richness was broadly consistent across disturbance levels with mean values ranging 43.4–51.8 per sampling unit (c. 3500 m^2^), with no statistically significant difference detected between REF and DEA in any of the pairwise comparisons (Fig. 3j). In contrast, heterogeneity diversity measures, exponential Shannon and inverse Simpson’s, were uniformly reduced in DEA relative to REF (all pairwise comparisons p < 0.05; Fig. 3k,l).

**Figure 4:**
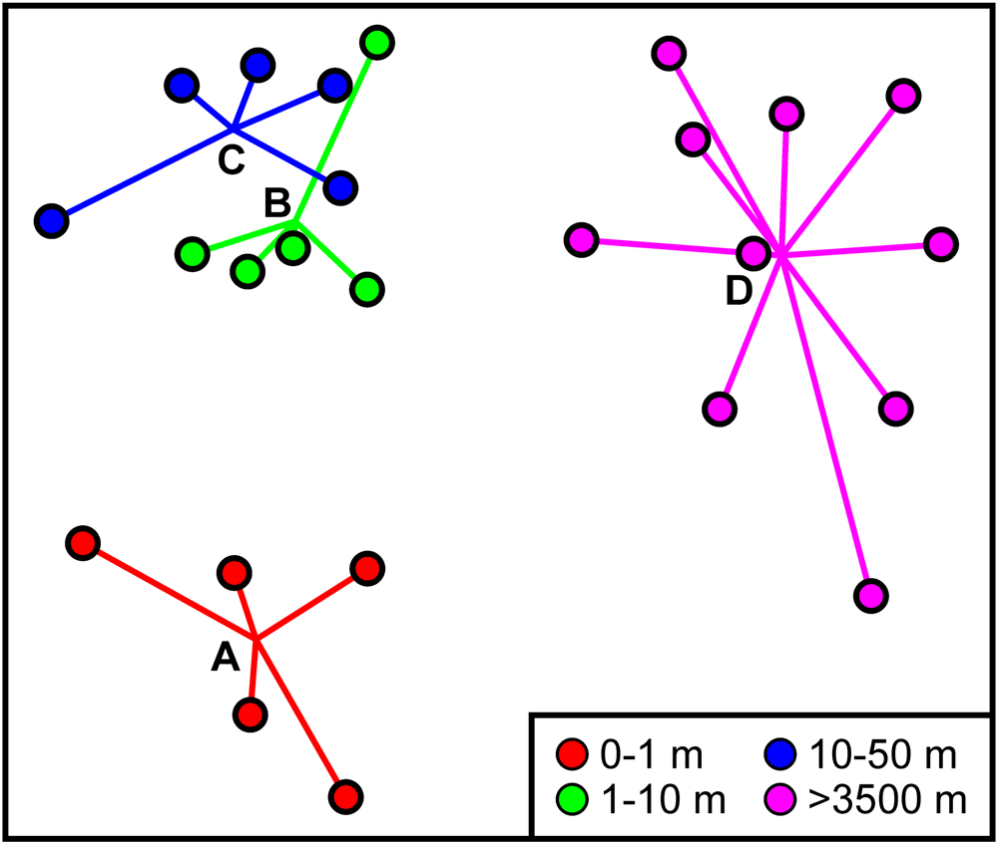
Variations in faunal composition by disturbance level. 2D non-metric multidimensional scaling ordination of Bray-Curtis dissimilarity based on square-root transformed taxon abundance data. Disturbance levels (A–D) are based on distance from plough tracks (key); symbols represent individual samples with lines linking to the centroid of replicates.

Ordination of faunal composition data (Fig. 4) readily distinguished DEA and REF samples, and plough track samples (level-A) from others within DEA. The y-ordinate of the ordination placed the DEA samples in presumed intensity of disturbance order, i.e. levels A-B-C, based on the centroids of the replicates. Formal comparison of faunal composition across the assemblages of different disturbance levels indicated a statistically significant difference overall (ANOSIM, R = 0.698, p < 0.0001) and statistically significant differences in all pairwise comparisons. All comparisons between DEA and REF were highly significant (pairwise ANOSIM R ≥ 0.705, p = 0.0003). Similarly, all comparisons between plough tracks (level-A) and other disturbance levels were highly significant (pairwise ANOSIM R ≥ 0.780, p ≤ 0.0079). The variation in faunal composition between level-B and level-C was statistically significant, though of modest magnitude (pairwise ANOSIM R = 0.304, p = 0.0317).

## Discussion

The effects of simulated deep-sea mining were clearly still evident in the physical character of the seabed and the associated megafaunal assemblage 26-years after the original disturbance of the DISCOL experimental area (DEA). Our results show statistically significant biological effects across the three primary seafloor categories: (i) direct physical disturbance in the plough tracks (PTs; disturbance level-A); (ii) indirect physical effects (sediment redeposition) in proximity to the PTs (disturbance levels-B-C); and (iii) the presumed undisturbed Southern reference area (REF). If we assumed that the REF area represents a “true control” for the disturbance experiment, we would conclude that the megabenthos of areas directly impacted by the plough-harrow and that of the adjacent areas only affected by sediment redeposition have not yet recovered. If we restrict our assessment to the DEA alone, then there is very clear evidence of continuing impact within the PTs, and some evidence of continuing impact in the immediate vicinity of the PTs (level-B).

### Standing stocks

**Figure 5:**
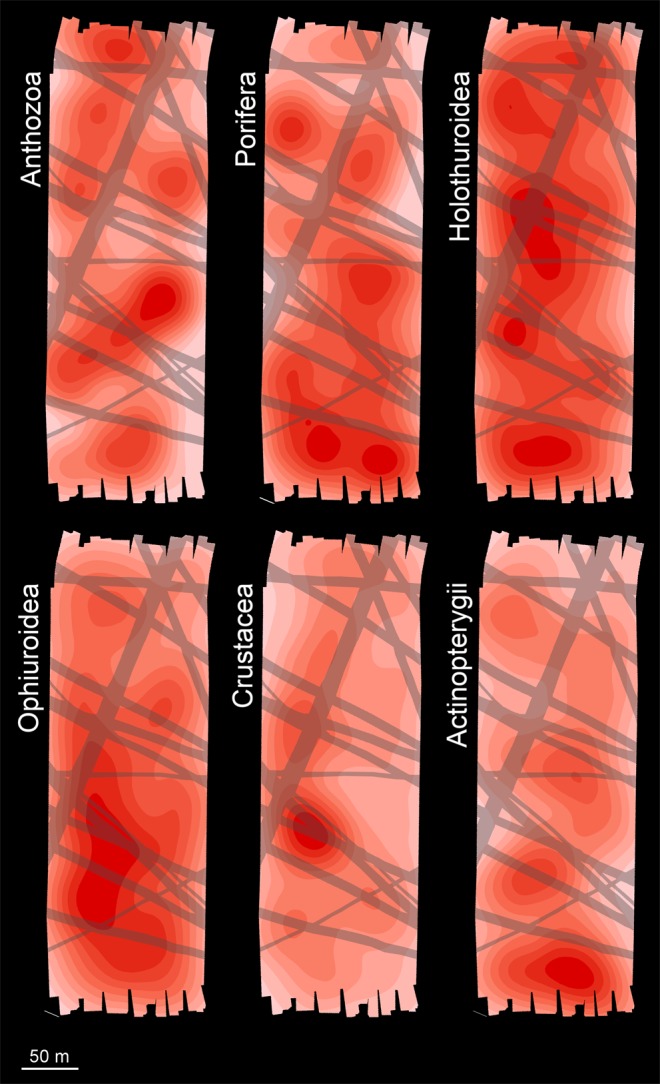
Heat maps showing the distribution of density of the six most-dominant megafaunal groups across the DEA. PTs depicted in semi-transparent stripes. Density ranges are classified in 10 equal breaks for each faunal group, from minimum (white) to maximum (red) density.

Differences in faunal density across the DEA were predominately driven by variations in suspension feeder abundance (Fig. 3i), particularly the Porifera and Anthozoa that were substantially reduced within PTs (Figs 3a,b and 5). Suspension feeder abundance is predominantly controlled by substratum availability in deep-sea ecosystems^28–30^, particularly in Pacific nodule fields^19,31^, where the populations are dominated by nodule-attached taxa^8,11^. Burial of nodules during the DISCOL experiment effectively eliminated this component of the local habitat, severely limiting the potential for recolonization by suspension feeders within PTs. Consequently, 26-years after the disturbance, suspension feeder standing stock remains substantially reduced within PTs, at 40% of that of other DEA and REF areas, as observed during previous DISCOL site revisits^24^. Suspension feeding organisms can provide a major contribution to the total faunal carbon in deep-sea benthic food webs^32^. These organisms can capture nutrient resources laterally transported across the benthic boundary layer, making them available for other organisms^33^. Substantial reductions of suspension feeding populations, in areas directly disturbed by nodule mining, are hence likely to generate a loss of key ecosystem functions.

The standing stocks of deposit feeders, and predators & scavengers, exhibited no statistically significant differences within the DEA (i.e. among levels-A-C) but in all cases were significantly enhanced relative to REF (Fig. 3g,h). Bluhm^24^ noted that repopulation of areas within PTs by deposit feeders and scavenging animals, with the exception of ophiuroids, started shortly after the original disturbance, almost reaching pre-disturbance levels after 7-years. Our data indicate that the numerical density of Crustacea, Holothuroidea, and Ophiuroidea exhibit little variation within DEA, and that Ophiuroidea are now more abundant than Holothuroidea, as was the case prior to the original disturbance^27^. The apparent enhancement of deposit feeder and predator & scavenger abundance in the DEA relative to REF, driven primarily by Ophiuroidea and Crustacea, has previously been noted for DEA areas outside PTs^24^. However, the causal factor is unclear. It may simply be a (subtle) difference in the physical environment between DEA and REF. Or potentially a broad-scale biological effect of the original disturbance, resulting from organic enrichment of surficial sediments (see e.g.)^34^ via mechanisms such as: redeposition of previously deeply buried organic matter, increased water-column organic matter ‘scavenging’ by the initially re-suspended sediment, or burial of formerly living biomass (i.e. mortality from original physical disturbance and sediment redeposition).

### Biological diversity

The heterogeneity diversity indices, exponential Shannon (exp[H′]) and inverse Simpson’s (1/D), both indicated no statistically significant differences within the DEA (levels-A-C), however, diversity was significantly lower in the DEA than the REF (Fig. 4k,l). The effect was strongest in Simpson’s index, η^2^ = 0.83 compared to 0.72 for the Shannon index (Table 1), suggesting a strong role for the dominance component of diversity. That dominance was driven by the relative abundance of Ophiuroidea, at 23% in REF and 34–36% in DEA areas. In other words, there was a lower megafauna taxa eveness in the disturbed areas of the DEA than in the presumably undisturbed seabed of the REF. To date, patterns in heterogeneity diversity have only been investigated for particular meio- and macrofaunal groups during DISCOL revisits, and have produced variable results. For example, 7-years after disturbance, heterogeneity diversity of the nematode assemblage was almost invariant across disturbance levels^26^, while the diversity of the of the polychate assemblage was still significantly reduced within PTs^25^. In contrast to the heterogeneity diversity metrics, variations in taxon richness with disturbance level were relatively modest (η^2^ = 0.38, Table 1), with mean values ranging from 43.4 (level-A) to 51.8 (level-C) taxa per sampling unit. Within the DEA there was a significant, though modest, decline in taxon richness in PTs relative to other DEA areas (levels-B-C). Note that the values displayed and tested here (Fig. 3j–l; Table 1) are in effect a measure of taxon density, the number of taxa per unit area (see e.g.)^35^. To a greater-or-lesser degree, all diversity measures are sensitive to the number of individuals within a sample, particularly in the case of taxon richness estimation^36^. Consequently, when comparing areas with varying faunal density, e.g. statistically significantly higher number of specimens per sampling unit in level-B and C compared to level-A and D, it is important to account for those variations^7,8^.

We examined the influence of number of specimens per sampling unit via rarefaction (see e.g.)^37^ as detailed in the supplement that accompanies the online publication. We concluded that at the employed physical sampling unit size (3500 m^2^), the corresponding range in specimen numbers per sampling unit within the DEA (199–300 individuals) had little or no impact on the interpretation of diversity results (Fig. A1). For example, when rarefied to a common number of individuals (1000 specimens), heterogeneity diversity was clearly reduced in DEA relative to REF, and taxon richness varied little across the four disturbance levels examined. Consequently, we consider that taxon richness or density shows no, or only very modest, variation with disturbance level, i.e. even within PTs taxon richness is or is becoming indistinguishable from background levels. Combining our observations with those of previous DISCOL studies, it appears that the reductions in diversity resulting from simulated mining disturbance can be long lasting (>26 years), however, detecting such patterns appears to be highly dependent on the faunal group studied (i.e. meio-, macro-, or megafauna) and the particular parameters used to investigate biological diversity^5^. Although megafaunal taxon richness may now show signs of recovery within PTs for the first time since the original disturbance^24^, the impacts of the DISCOL ploughing are still very evident in the composition of the fauna.

### Community composition

Our analyses indicate statistically significant differences in faunal composition between all four disturbance levels considered. Those differences were very substantial between all DEA and REF comparisons (ANOSIM R ≥ 0.705) and between plough tracks and other DEA areas (levels-B-C; R ≥ 0.780) (Fig. 4).

We have considered the potential drivers of enhanced deposit feeder abundance (e.g. Ophiuroidea in DEA) and reduced suspension feeder abundance (e.g. Porifera and Alcyonacea in DEA and particularly PTs) in the standing stocks section, suggesting potential organic enrichment and loss of nodule habitat as causes. The statistically significant increase in the numerical density of fish in level-B and C areas relative to the PTs and REF, exemplified by *Ipnops* sp. that represents 88% of 324 fish encountered in the present study, was a somewhat unexpected result. As highly mobile organisms, fish are perhaps unlikely candidates as indicators of change in an open sedimentary abyssal habitat, nevertheless, *Ipnops* sp. is likely to be an important benthic and benthic boundary layer predator in this environment^38,39^. As total megafauna numerical density was significantly enhanced in level-B and C areas of the DEA relative to the PTs and REF (Table 1) it may not be surprising that a key predator follows the same trend.

**Figure 6:**
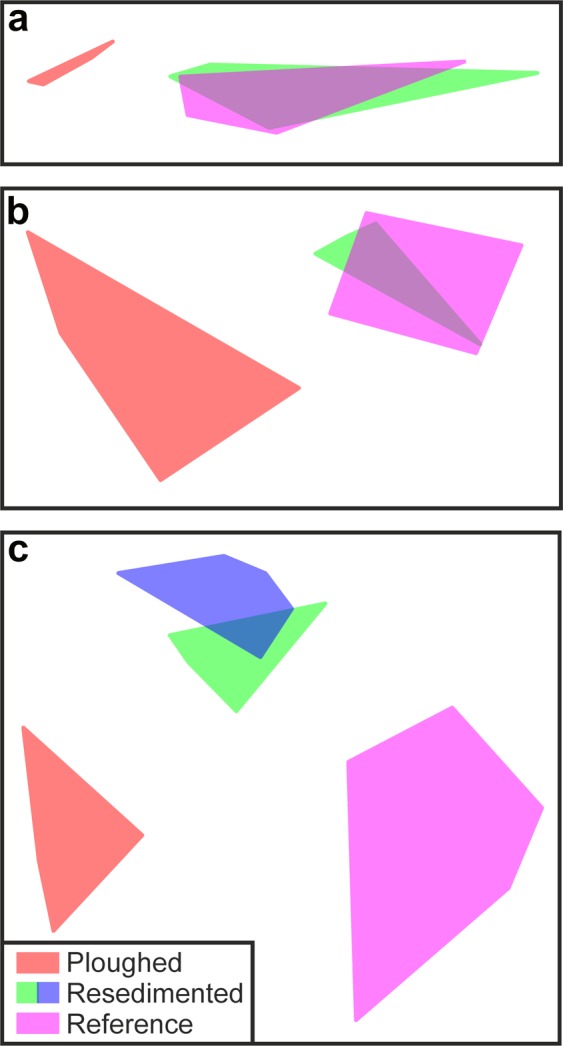
Comparison of assessments of variations in megafaunal composition with disturbance type in DISCOL studies. (**a**) Bluhm^24^ euclidean dissimilarity, no faunal density transformation. (**b**) Bluhm^24^ cosine dissimilarity, no faunal density transformation. (**c**) Present study Bray-Curtis dissimilarity, square-root transformation of faunal density. Note that present study recognises two classes of resedimented area (disturbance levels B and C).

A number of factors complicate the comparison of our results with those of prior DISCOL studies: limited or no replication, methodological variations, and some variation in the selection or use of ‘reference’ areas. The closest match to the present study is the comprehensive assessment of megafauna provided by Bluhm^24^ that covers the time-period immediately pre-disturbance to 7-years post disturbance. We summarise Bluhm’s multivariate analyses with our own in Fig. 6. Subjective assessment of the earlier work suggests a clear distinction of plough tracks, but no distinction of resedimented areas from reference conditions (Fig. 6a,b). In contrast, our study suggested a clear distinction of plough tracks, resedimented, and reference areas (Fig. 6c).

Patterns in community composition have been investigated for samller faunal groups (meio-, and macrofauna) in previous DISCOL revisits producing variable results. Borowski^25^ provides a comparable assessment of macrobenthos, however, no formal testing of variations in faunal composition is presented; the author notes that major differences in macrofauna higher taxa, polychaete families, or polychaete species were not detected between ploughed, resedimented, and reference sites. Nevertheless, Borowski^25^ does record that a scavenger/predator sigalionid polychaete species, *Leanira* sp. A, showed a repeated trend of high abundance in the resedimented areas relative to PTs and reference areas, suggesting this as a possible response to enhanced food availability for this species. There have also been studies of the meiobenthos that variously report differences in faunal composition between ploughed and resedimented areas (nematodes^26,40^; harpacticoids^23^), however, they do not make comparisons with reference areas. The results of these studies, along with those obtained in this contribution, support that organisms of different sizes and functional groups may have a different sensitivity to the impacts of simulated mining^5^, being suspension feeding megafauna one of the functional groups that exhibits the clearest responses.

### Relevance to commercial nodule mining

Ecological assessments of the effects of simulated nodule mining on the megabenthos of the Clarion-Clipperton Zone (CCZ)^19,31^ have yielded similar results to those obtained in the Peru Basin^24,27^ (this study). Suspension feeders, particularly Anthozoa, consistently showed the highest sensitivity to impacts, exhibiting substantial reductions in standing stock, both in the short and long term, after disturbance^5^. The characterisation of post-disturbance biological responses described in these studies are important to further define “serious harm”, a key concept for the effective regulation of nodule mining activities^1^. However, nodule mining impact simulations performed to date do not mimic the full range or magnitude of disturbances expected from commercial mining (e.g. sediment compaction, surface sediment layer removal, release of toxic elements, etc.), nor their likely spatial extent^12^. Moreover, baseline assessments have shown that the relative proportion of suspension feeders in some CCZ areas, where commercial mining is most likely, is generally much higher than encountered in the Peru Basin^19^. In the CCZ, nodule-attached Anthozoa and Porifera often dominate the megabenthic community^7,11^. Consequently, commercial-scale mining in the CCZ may exert a greater impact on the structure and function of megabenthic assemblages than we have observed in the DISCOL plough tracks (see e.g.)^41^.

It seems clear that we still do not have a good understanding of the impact of sediment redeposition beyond plough tracks. The present study suggests that even 26-years post-disturbance, the area of redeposition remains ecologically distinct – in standing stock, biological diversity, and faunal composition – from the reference area. Without such knowledge, it will be difficult to gauge the true extent or recovery timescale for the cumulative mining disturbances that may affect nodule-fields at the regional scale. Obtaining that knowledge depends upon selecting and monitoring appropriate control (reference) sites. Indeed that is a weakness of the present study, in that we cannot be entirely certain that the Southern reference area is an entirely appropriate control for the DEA. This lack of representivity of control sites may also be an issue for management and monitoring of commercial mining, which can be addressed, in part, by careful baseline assessment^42^. Nevertheless, the need for control sites is clear and must form a key criterion for the selection of “Areas of Particular Environmental Importance” (APEIs) in the CCZ^43^ and ocean observatory sites more generally (see e.g. the Deep Ocean Observing Strategy, deepoceanobserving.org). Given that, it may ultimately be impossible to establish ‘perfect’ controls of the necessary physical scale, a gradient approach to the assessment of the diffused effects of mining, i.e. similar to what we have attempted here, may also be valuable in assessing the impact of redeposited sediments.

Last, it is important to note that the assessment performed in this study was the result of just two AUV deployments. This shows that comprehensive AUV-based survey designs have a great potential to aid the assessment of disturbance, while reducing ship-time, in mining-impact monitoring. Also the 2d-approach (photo-mosaic) allowed a graded assessment of disturbance, not readily possible from prior towed-camera efforts which typically follow long tracks. Thus we see the presented photo-mosaic approach as an effective tool for evaluating megabenthic distribution patterns in disturbed and reference sites prior and after impact. The methodology and workflow used here should be considered as approach in similar investigations to come.

## Methods

### Study area

All data were acquired during RV *Sonne* expedition SO242-1 to the DISCOL site, 07° 06′S - 088°27′ W, in the Peru Basin^44^ (Fig. 1a). The seafloor landscape in the DISCOL area ranges from 3800 to 4300 m water depth and is characterized by a succession of crenulated hills and shallow troughs between dispersed level-bottom (<5° slope) area (Fig. 1b). Typical oceanographic characteristics are: bottom water temperature 1.8 °C, salinity 34.6 PSU, oxygen concentration 139 μM L^−1^ ^44^. Bottom water currents are relatively modest (<15 cm s^−1^) with residual flow to the northwest (310°)^45^. Surface sediments have an average total organic carbon content (C_org_) of 0.64% and are typically composed by silty clays or clayey silts (Clay: 65%; Silt: 20%; Sand: 15%) with little regional variation^46,47^. The DEA and the Southern reference area (REF) show similar environmental features. Both areas are located on a relatively smooth, slightly elevated part of the DISCOL seafloor, where water depths range from 4140 to 4160 m (Fig. 1b), and polymetallic nodule mass density averages >10 kg m^−2^, although it may reach 30–40 kg m^−2^ locally^21,44^.

### Seafloor imaging

We conducted an AUV-based visual survey of the seafloor in the centre of the DEA (88.465°W 7.074°S) and in the REF (88.450°W 7.126°S). The GEOMAR AUV Abyss (REMUS 6000, Hydroid, Inc.), was equipped with a Canon EOS-6D camera, a 15 mm fisheye objective lens, and a cluster of 24 LED lighting arrays^48^. The vehicle was programmed to fly approximately 4.5–5 m above the seafloor at a speed of 1.5 m s^−1^, recording a seafloor image every second, along a series of parallel transects, to generate a 100% seabed mapping^48^. Imagery was curated as described in^49^, full station details and image data analysed during the current study are available in the PANGAEA repository, 10.1594/PANGAEA.881850. The resultant mosaics were processed to a common seabed resolution of 3 mm pixel^−1^, having a final seafloor extent of 5.86 ha in the DEA, and 5.25 ha in the REF area (Fig. 1c,e).

### Seafloor characterisation

**Figure 7:**
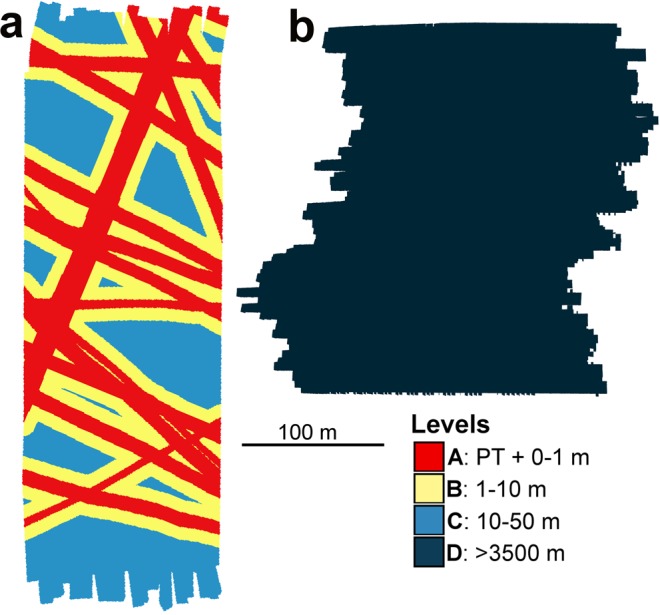
Seafloor disturbance classification. (**a**) DISCOL Experimental Area (DEA) seafloor mosaic. (**b**) Southern reference area (REF) seafloor mosaic. Disturbance levels (A–D) are classified as horizontal distance from visible plough tracks.

The 26-year-old original DISCOL plough tracks appeared to be readily identifiable in the AUV seafloor images (see e.g. Fig. 1c,d) and their positioning matched with that detected in sidescan sonar data also collected during SO242-1^44^. Consequently, we were able to visually classify the seafloor into three categories: (i) Physically disturbed areas, i.e. PTs in DEA; (ii) Areas apparently subject to sediment redeposition, i.e. adjacent to, and beyond, PTs in DEA; and (iii) Apparently undisturbed areas, i.e. all of REF site. For formal analyses we defined seabed areas by their proximity to the identifiable PTs. A 0.5 m resolution raster was produced for the DEA site image mosaic recording the horizontal distance to the nearest PT. Each cell of the raster was then classified into one of three disturbance levels based on PT proximity: level-A, 0–1 m; level-B, 1–10 m; and level-C: 10–50 m (Fig. 7a). The full extent of the REF image mosaic was classified as level-D, > 3500 m from PT (Fig. 7b).

### Megafauna assessment

The image mosaics were divided into sets of georeferenced tiles, each tile representing approximately 10 m^2^ of seafloor. The tiles were then analysed in random order to minimise any sequence- or time-related bias (e.g.)^50^. The tiling procedure was undertaken as a practical measure to simplify handling of the otherwise extremely large image, and to improve assurance of full coverage analysis and avoid the risk of double counting individual specimens. The metazoan megafauna specimens encountered during the analysis of the tiles were identified to the lowest taxonomic level possible (morphospecies: msp), and measured using the BIIGLE 2.0 image annotation software system^51^. To ensure consistency in specimen identification, a standardised megafauna catalogue was developed based upon existing megafauna compilations (http://ccfzatlas.com; https://www.discol.de/megafauna), which we expanded in consultation with taxonomic experts and by reference to existing literature. The likely feeding behaviour of each morphospecies was inferred from similar organisms described in the literature. For internal consistency and to enable definitive future comparisons, only those specimens with a dimension >5 cm were included in the subsequent analyses. The choice of a 5 cm minimum dimension was based on the seabed image resolution achieved (3 mm pixel^−1^) and reflects the relatively high operating altitude of the AUV (4.5–5 m). To enable referencing to seabed disturbance levels, the geolocation of each specimen was estimated from the AUV navigation data and the location of the specimen within the tile. A total of 7284 metazoan individuals (>5 cm) were recorded in the 11.1 ha of seabed examined during the present study (Supplementary Table S1). Megafauna specimens were classified into 97 morphospecies and 10 higher taxonomic categories (i.e. Order, Family).

### Selected taxon assessment

The original assessments of the DISCOL megafauna reported by Bluhm^14,27^ examined 11 dominant taxonomic groups of identifiable specimens. From those, we selected the six most abundant groups in the present study, specifically those having over 100 records from both the DEA and the REF mosaic areas. The selected taxa were: Anthozoa, Porifera, Holothuroidea, Ophiuroidea, Crustacea, and Actinopterygii (i.e. Osteichthyes in)^14,27^ (Fig. 3a–f). Note that Anthozoa includes Bluhm’s original categories Actiniaria, and taxa from their ‘Rest’ category (Pennatularia [=Pennatulacea], Gorgonaria [=Alcyonacea], Ceriantharia, and Antipatharia). Heat maps illustrating the spatial distribution of these six groups across the DEA mosaic were generated at 1-metre raster resolution based on a circular neighbourhood radius of 20 metres, using the ‘kernel density’ tool in ArcMap v10.2^52^. Formal testing of variations in taxon group density with disturbance level was as detailed in the full assemblage assessment section below.

### Full assemblage assessment

To carry out the assessment of variations in ecological metrics with disturbance level we conducted an *a posteriori* stratified random sampling scheme (see e.g.)^53^. Each photo-mosaic, and the faunal data associated, was first converted to a 0.5 m seabed resolution raster and each cell classified to disturbance level (see Seafloor Characterisation section). Composite sampling units of 14000 cells (3500 m^2^) were then formed by random allocation within each disturbance level. By this process we generated five replicate sampling units each for disturbance levels A, B, and C (the DEA site photo-mosaic) and ten replicate sampling units for disturbance level D (“no disturbance”, Southern reference area photo-mosaic, REF). Note that this process effectively destroyed the spatial coherence of the data by randomisation -minimising the inevitable occurrence of spatial autocorrelation between adjacent tiles (e.g.)^54^- and pooled the biological data into sampling units of sufficient size to calculate useful faunal diversity and similarity values. Each replicate having a physical scale of 3500 m^2^ and encompassing 197–299 faunal individuals.

A range of ecological parameters were calculated for each of these replicates: numerical density, expressed as individuals per hectare (ind ha^−1^); and Hill’s diversity numbers of order 0, 1, and 2^55^; respectively morphospecies richness (S), the exponential form of the Shannon index (exp[H′]), and the inverse form of Simpson’s index (1/D), expressed as number of taxa per sampling unit. Each order reflects an increasing sensitivity to the relative abundance of different taxa (evenness) in the assessment of diversity^55,56^. Diversity values were calculated using functions provided in the ‘vegan’ package implemented in R^57^. Additional assessments of diversity, examining the effect of sampling unit size, were also carried out as detailed in the supplement that accompanies the online publication.

Statistical comparisons of variations in taxon density and diversity by disturbance level were carried out using generalized linear models (GLM)^58^, as implemented in the ‘car’ package in R^59^. Homogeneity of variance and probability-distribution assumptions were verified by visual inspection of model histograms and QQ plots. Variations in faunal density were tested based on (integer) abundance count data, as all sampling units covered the same seafloor area. Models were fitted with quasi-Poisson errors for abundance metrics with over-dispersion^60^, and with Gaussian errors for diversity metrics^61^. Where statistically significant differences were detected in these global tests, simultaneous tests were applied to make multiple comparisons between individual disturbance levels using the ‘multcomp’ package in R^62^, p-value adjustments for multiplicity were made using the ‘mvt’ single-step procedure^63^. The effect size measure η^2^ (eta-squared)^64^; was also calculated using the ‘sjstats’ package in R^65^.

Variations in community composition between disturbance levels were explored following an abundance-based multivariate approach. Dissimilarity in faunal composition between all pairs of replicate samples was calculated using the Bray-Curtis dissimilarity measure, based on square-root transformed faunal abundance. Non-metric multidimensional scaling (nMDS) ordination was used to visualise potential variations in taxa composition between replicate samples representing each disturbance level. A one-way analysis of similarities (ANOSIM) with follow-up pairwise tests was used to assess variations in assemblage composition between disturbance levels. All multivariate analyses were implemented using the software package PRIMER v.7^66^.

## Supplementary information

Supplementary material
