# Supplementary material for: Biological effects 26 years after simulated deep-sea mining
Source: Sci Rep. 2019 May 29;9:8040. doi: 10.1038/s41598-019-44492-w (PMC6541615; doi:10.1038/s41598-019-44492-w)
Supplement: Supplementary file 1 — Supplementary material [file 41598_2019_44492_MOESM1_ESM.pdf]

Biological effects 26 years after simulated deep-sea mining

Erik Simon-Lledó[1,2,*], Brian J. Bett[1], Veerle A.I. Huvenne[1], Kevin Köser, Timm Schoening[3], Jens Greinert[3-4], Daniel O.B. Jones[1]

[1]National Oceanography Centre, Empress Dock, Southampton SO14 3ZH, UK
[2]Ocean and Earth Science, University of Southampton, Southampton SO14 3ZH, UK
[3]GEOMAR Helmholtz Centre for Ocean Research Kiel, D-24148 Kiel, Germany
[4]Christian-Albrechts University Kiel, Institute of Geosciences; D-24098 Kiel, Germany
*** Correspondence:** erimon@noc.ac.uk

Supplementary material

Table S1. Total abundance and taxon richness of major taxa encountered during the present study. DEA, DISCOL experimental area photo-mosaic, survey area 5.86 ha. REF, southern reference area photo-mosaic, survey area 5.25 ha.

Phylum	Class	Order	Morphospecies	DEA	REF
**Porifera**					
	Demospongiae		4	49	100
	Hexactinellida		7	215	206
**Ctenophora**					
	Tentaculata		2	34	28
**Cnidaria**					
	Anthozoa				
		Actiniaria	7	106	208
		Alcyonacea	3	39	130
		Antipatharia	2	11	7
		Ceriantharia	1	2	0
		Pennatulacea	1	2	2
		Zoantharia	1	10	7
	Hydrozoa		2	107	60
**Bryozoa**					
	Gymnolaemata		1	2	1
**Annelida**					
	Polychaeta		4	112	88
**Arthropoda**					
	Malacostraca				
		Decapoda	6	407	305
		Isopoda	1	7	2
		Cirripedia	1	2	2
**Echinodermata**					
	Asteroidea		7	90	62
	Crinoidea		5	125	94
	Echinoidea		2	9	2
	Holothuroidea		26	992	794
	Ophiuroidea		2	1409	724
**Hemichordata**					
	Enteropneusta		2	82	77
**Mollusca**					
	Cephalopoda		3	13	32
**Chordata**					
	Ascidiacea		1	94	60
	Actinopterygii		6	233	141

Supplementary analysis

Effect of sampling unit size on the interpretation of diversity metrics

We applied a rarefaction approach to assess the potential impact of sampling unit size on the interpretation of calculated values of taxon richness, the exponential Shannon index (exp[H']), and the inverse Simpson's index (1/D) [1]. Sampling unit size was quantified as both number of individuals and seabed area observed. Faunal data (in raster cells: 0.5 m resolution; see main text) was collated for each disturbance level and randomly resampled 1000 times, with (Exp[H'], 1/D) or without (taxon richness) replacement, to form increasingly larger sampling units. The mean and 95% confidence intervals of each parameter were calculated at each sampling unit size. Taxon richness assessment was implemented using Estimate S v.9.1 software [2], while exp[H´] and 1/D were computed using a custom in R accessing the 'vegan' package.

Taxon richness curves showed no significant variations between disturbance levels in sample sizes up to 1000 individuals (Fig. S1a). Shannon diversity in REF (level-D) was clearly elevated above all three DEA areas at samples sizes over c. 550 individuals (Fig. S1d). Similarly, Simpson's index in REF (level-D) was clearly elevated above all three DEA areas at samples sizes over c. 350 individuals (Fig. S1f).

Reference

1 Chao, A. *et al.* Rarefaction and extrapolation with Hill numbers: a framework for sampling and estimation in species diversity studies. *Ecological Monographs* **84**, 45-67, doi:doi:10.1890/13-0133.1 (2014).

2 Colwell, R. EstimateS: Statistical estimation of species richness and shared species from samples. Version 9. *User's Guide and Application published at: http://purloclcorg/estimates* (2013).

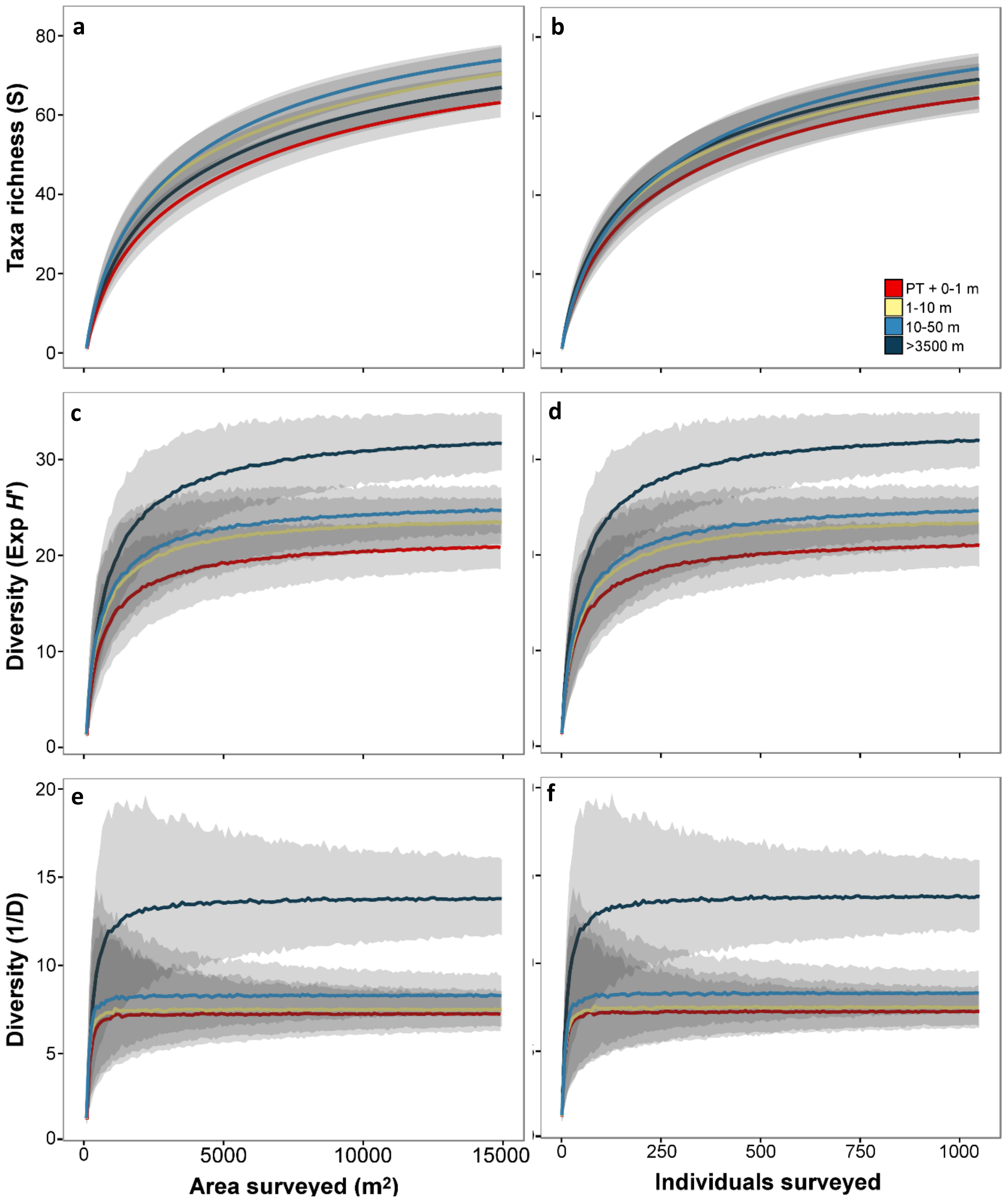

Figure S1. Variation in faunal diversity as a function of sampling unit size for each disturbance level assessed. Lines represent mean value of 1000 randomisations, shaded area represents corresponding 95% confidence interval. **a-b:** Rarefied morphospecies accumulation curves. **c-d:** Variation in Exp (H') diversity. **e-f:** Variation in 1/D diversity.
